# Supplementary figures and images for: Distinct states of nucleolar stress induced by anticancer drugs
Source: eLife. 2023 Dec 15;12:RP88799. doi: 10.7554/eLife.88799 (PMC10723795; doi:10.7554/eLife.88799)

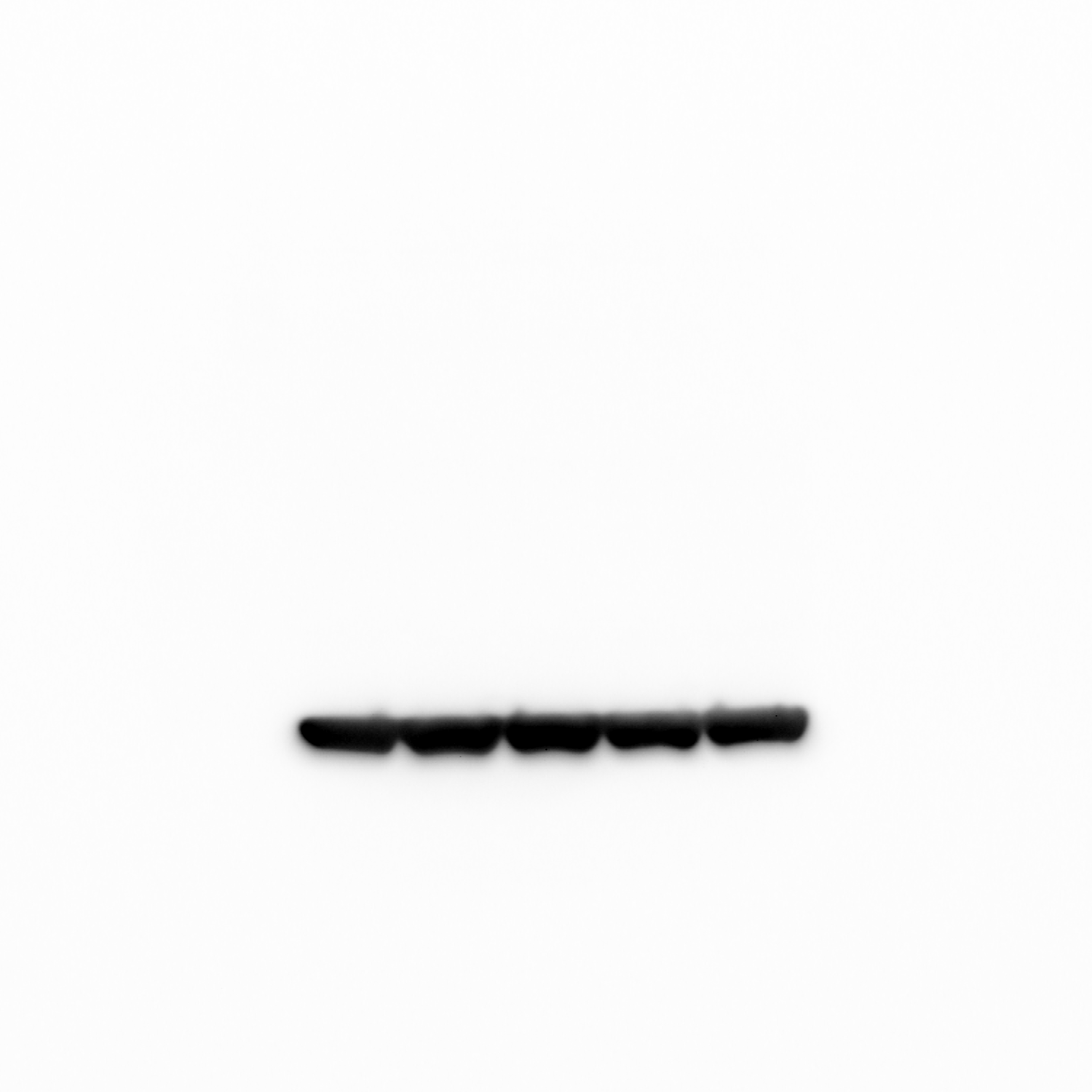

Supplement: Figure 3—source data 2. [file elife-88799-fig3-data2.zip › Figure 3-source data2/3F/POLR1A _beta actin.tif]

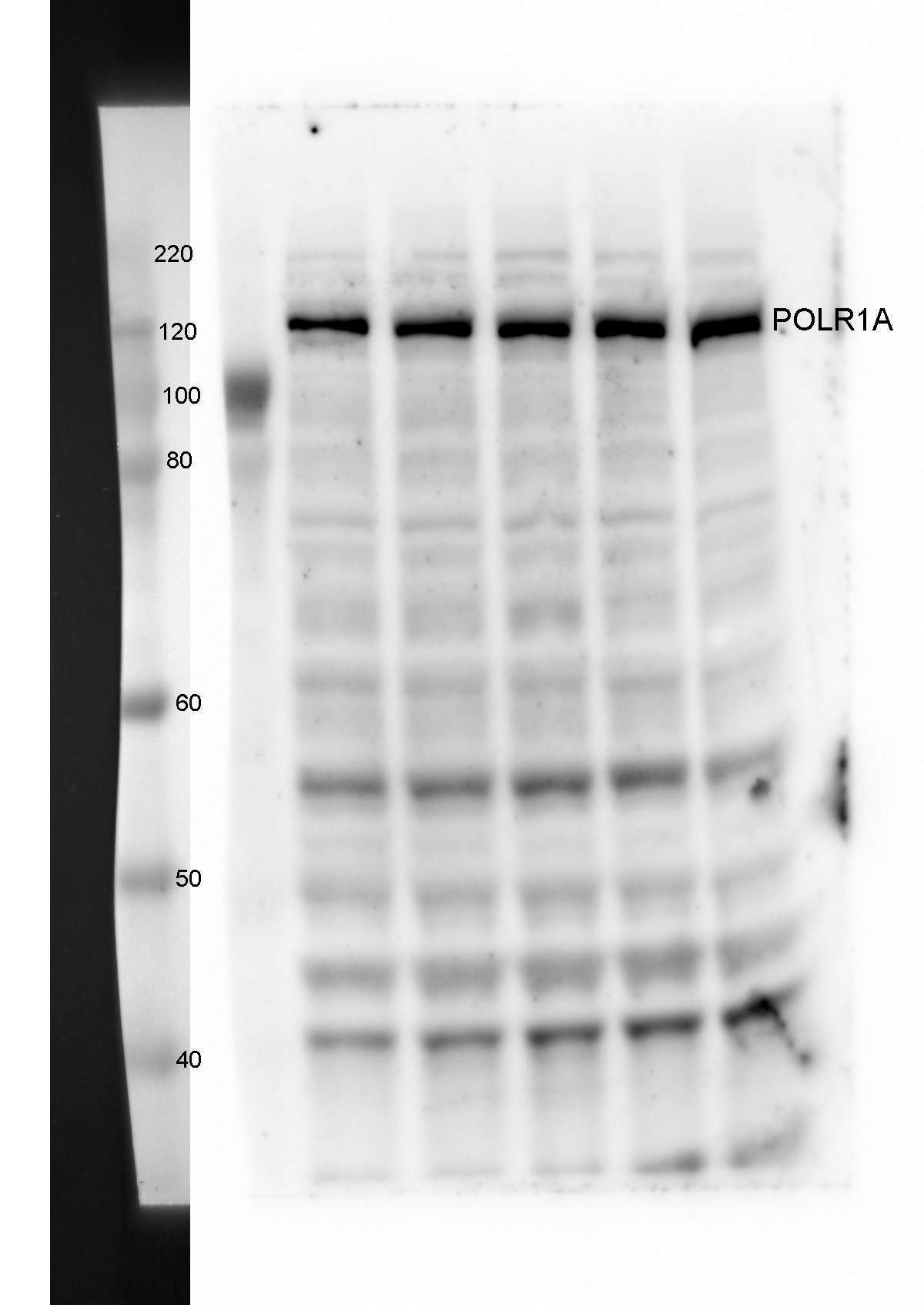

Supplement: Figure 3—source data 2. [file elife-88799-fig3-data2.zip › Figure 3-source data2/3F/POLR1A blot_annotated.jpg]

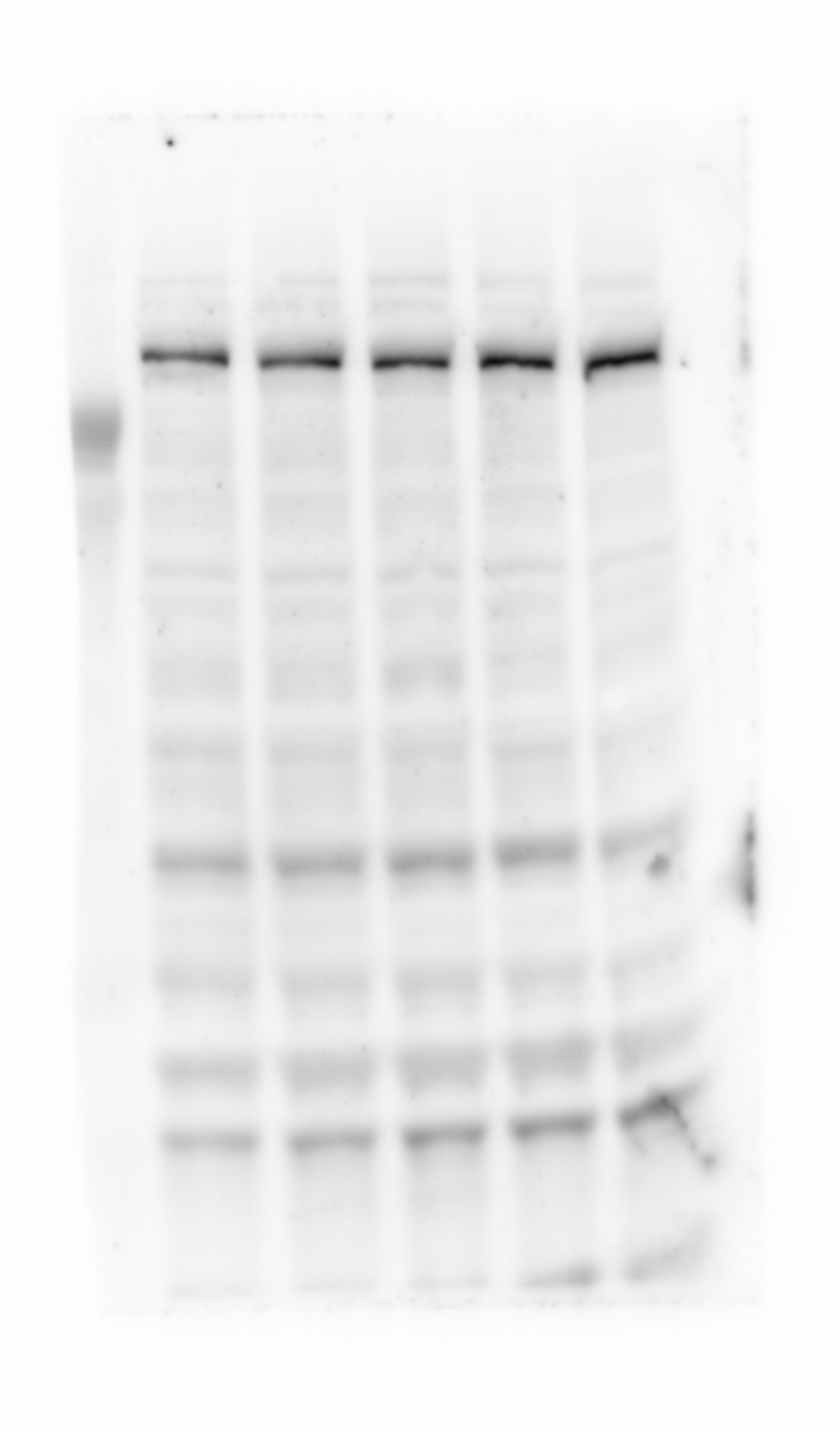

Supplement: Figure 3—source data 2. [file elife-88799-fig3-data2.zip › Figure 3-source data2/3F/POLR1A_20sec_1.tif]

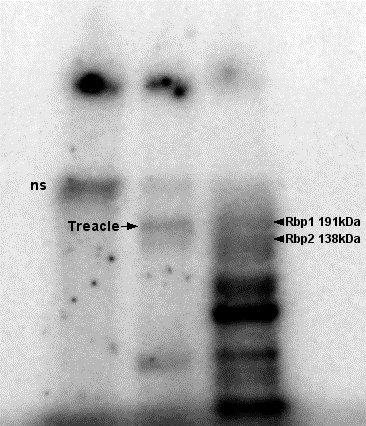

Supplement: Figure 5—source data 1. [file elife-88799-fig5-data1.zip › Figure 5-source data1/Figure 5-source data 5D/CDK9 assay with Treacle annotated.jpg]

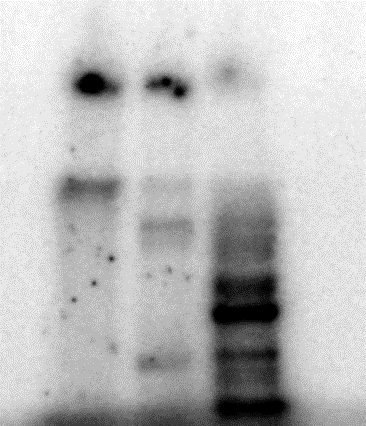

Supplement: Figure 5—source data 1. [file elife-88799-fig5-data1.zip › Figure 5-source data1/Figure 5-source data 5D/CDK9 assay with Treacle.tiff]

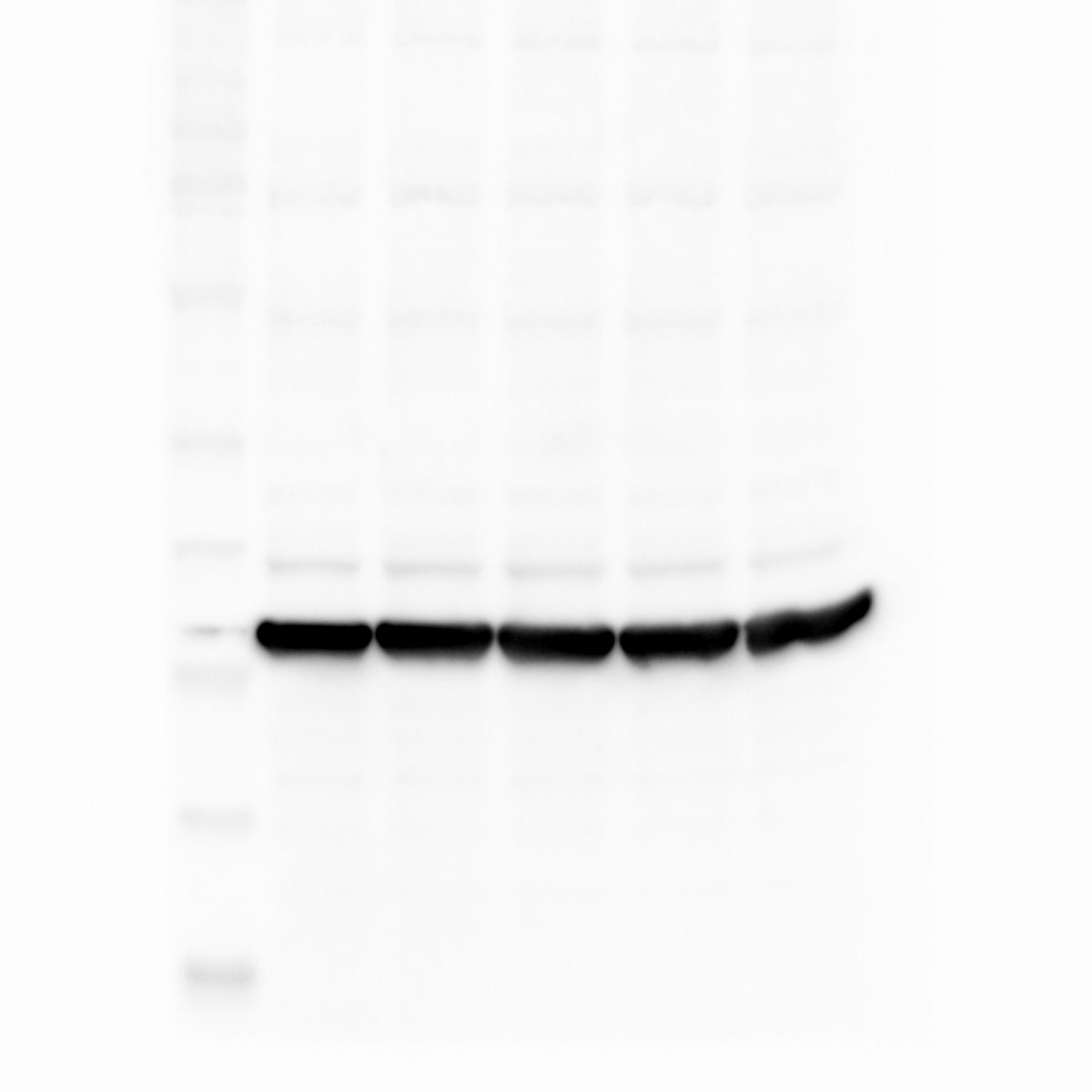

Supplement: Figure 5—source data 2. [file elife-88799-fig5-data2.zip › Figure 5-source data2/Figure 5-source data 5E/Beta-actin 15 sec_1 TCOF IP WCL.tif]

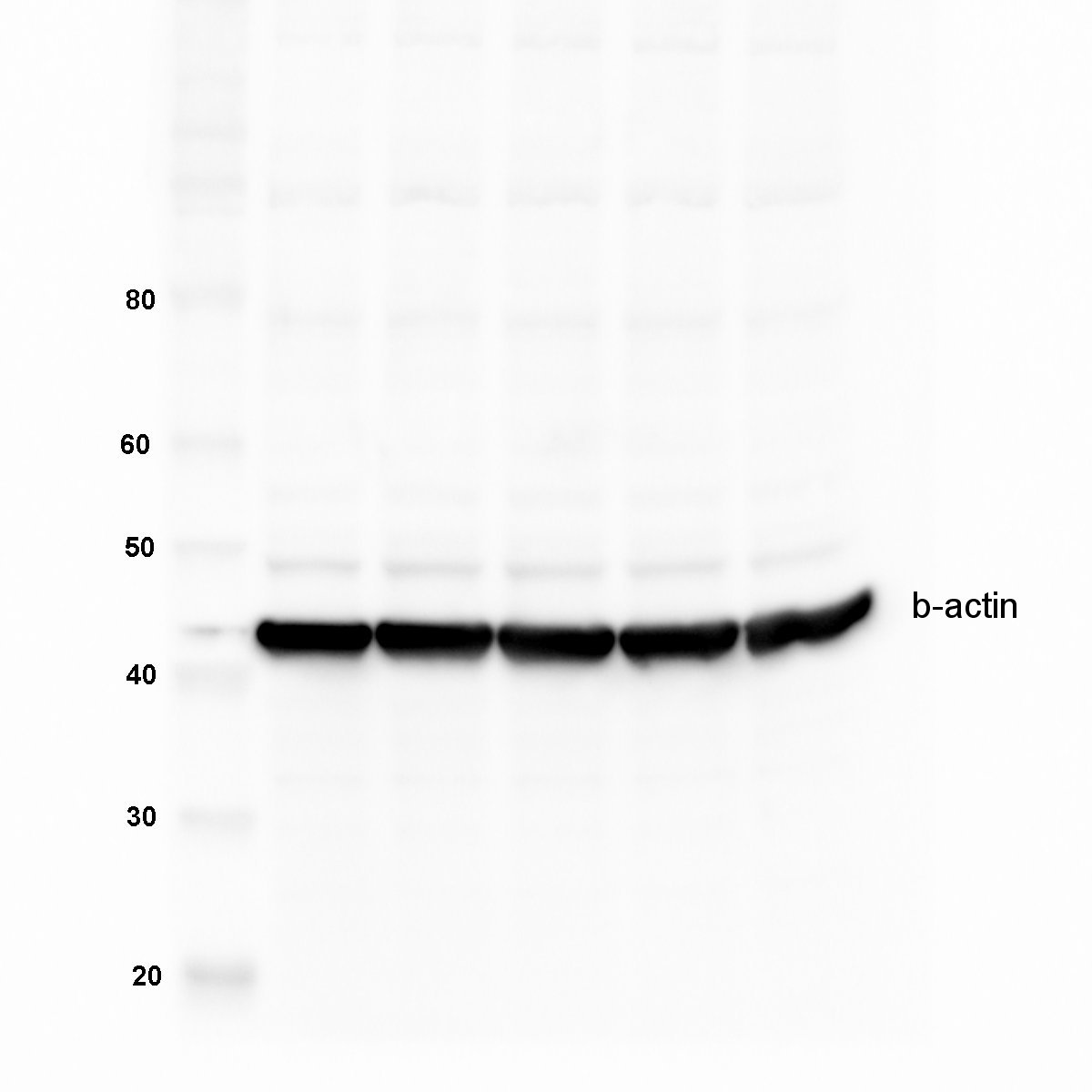

Supplement: Figure 5—source data 2. [file elife-88799-fig5-data2.zip › Figure 5-source data2/Figure 5-source data 5E/Beta-actin 15 sec_1 TCOF IP WCL_annotated.jpg]

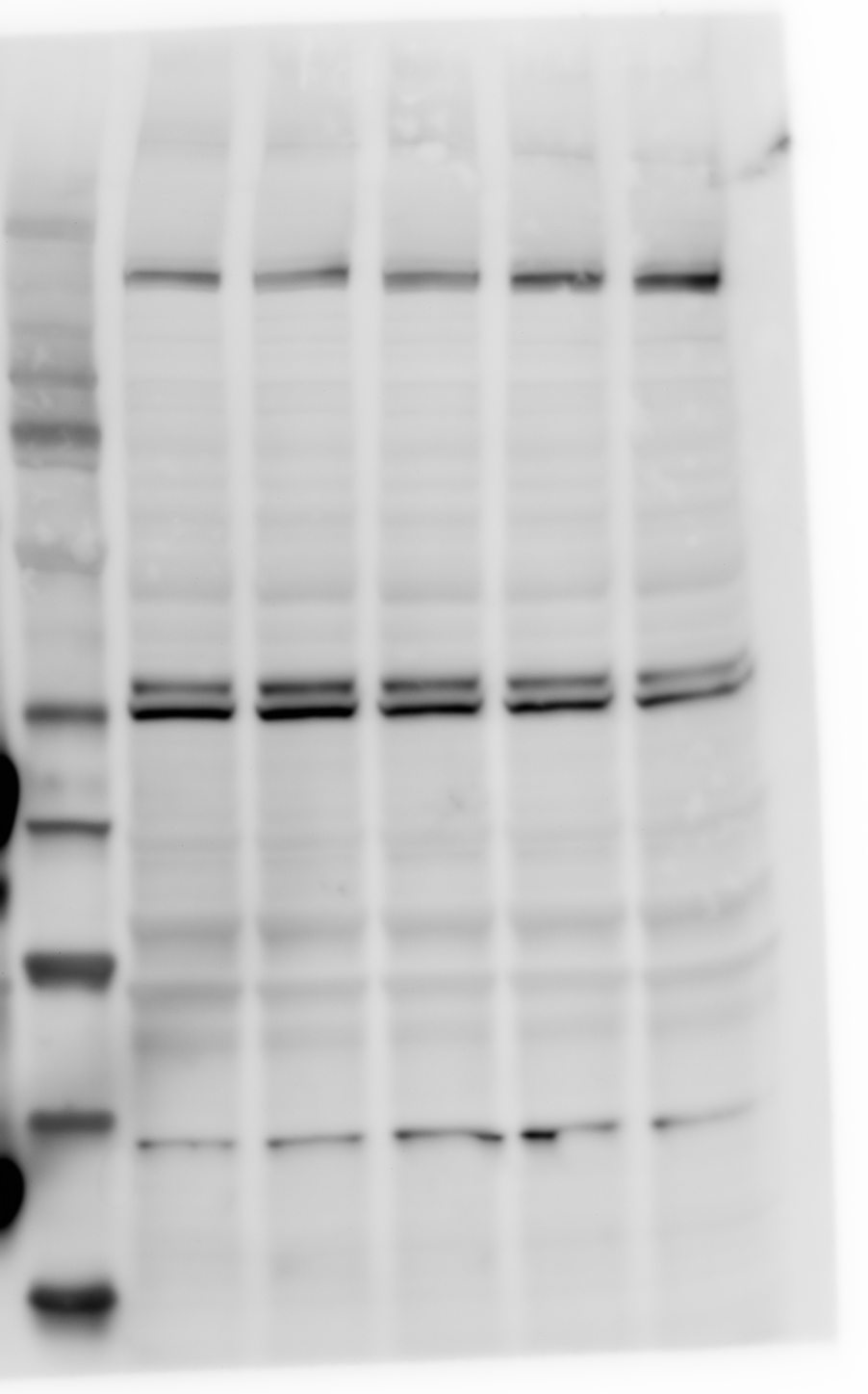

Supplement: Figure 5—source data 2. [file elife-88799-fig5-data2.zip › Figure 5-source data2/Figure 5-source data 5E/POLR1A IP TCOF1 WCL blot 2min.tif]

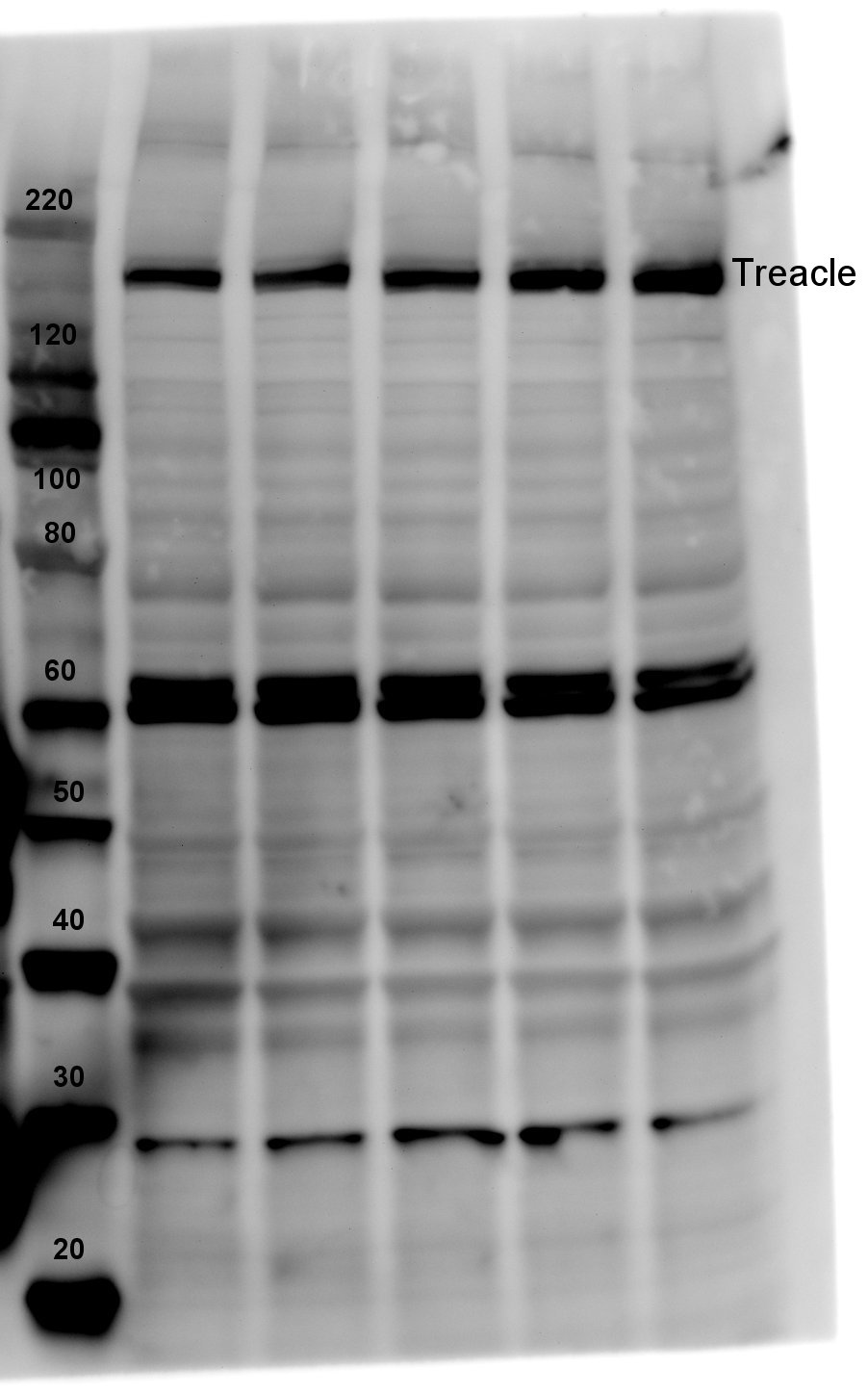

Supplement: Figure 5—source data 2. [file elife-88799-fig5-data2.zip › Figure 5-source data2/Figure 5-source data 5E/POLR1A IP TCOF1 WCL blot 2min_annotated.jpg]

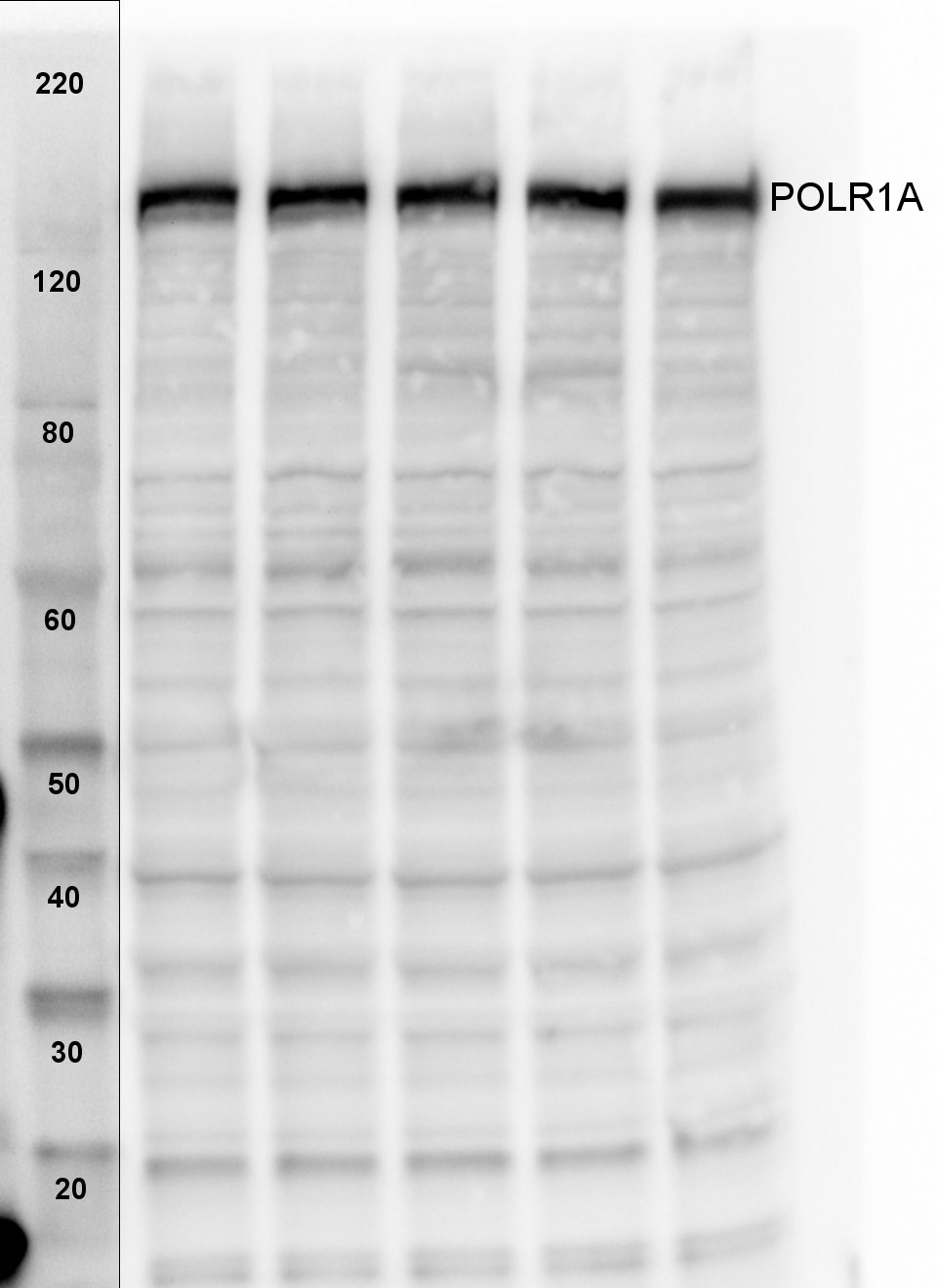

Supplement: Figure 5—source data 2. [file elife-88799-fig5-data2.zip › Figure 5-source data2/Figure 5-source data 5E/TCOF IP POLR1A WCL blot 30sec annotated.jpg]

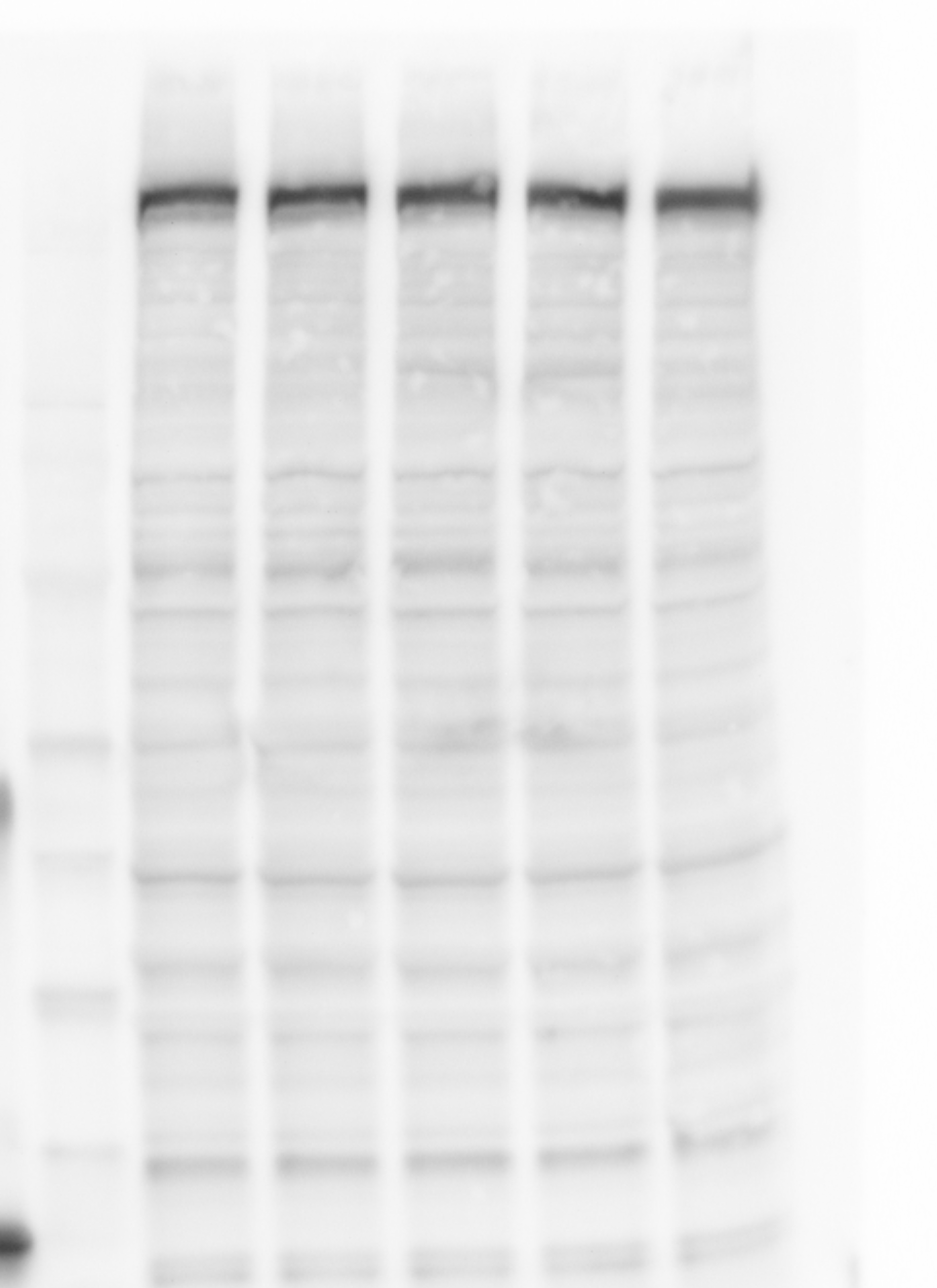

Supplement: Figure 5—source data 2. [file elife-88799-fig5-data2.zip › Figure 5-source data2/Figure 5-source data 5E/TCOF IP POLR1A WCL blot 30sec.tif]

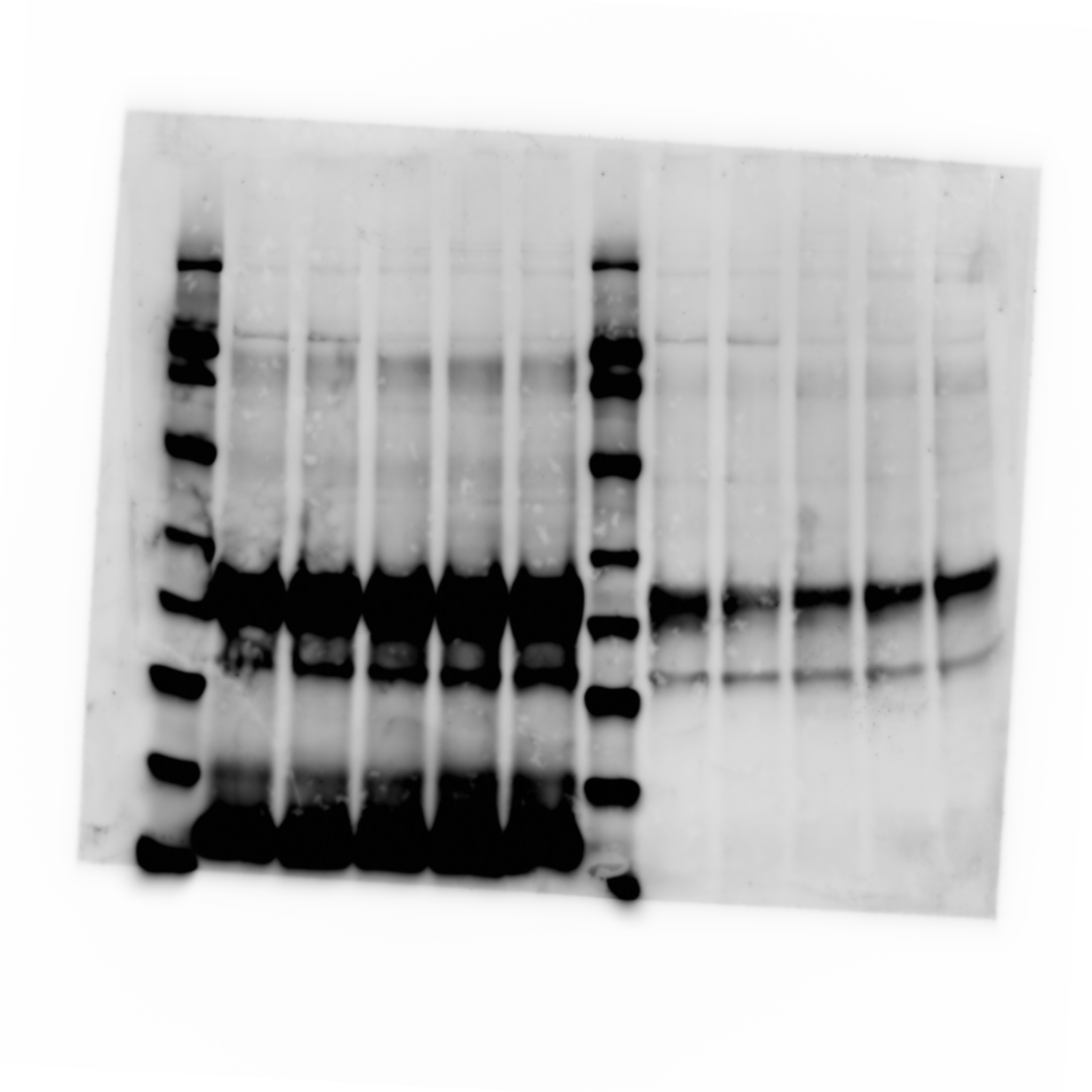

Supplement: Figure 5—source data 2. [file elife-88799-fig5-data2.zip › Figure 5-source data2/Figure 5-source data 5E/TCOF IPs POLR1A blot 2.5 min.tif]

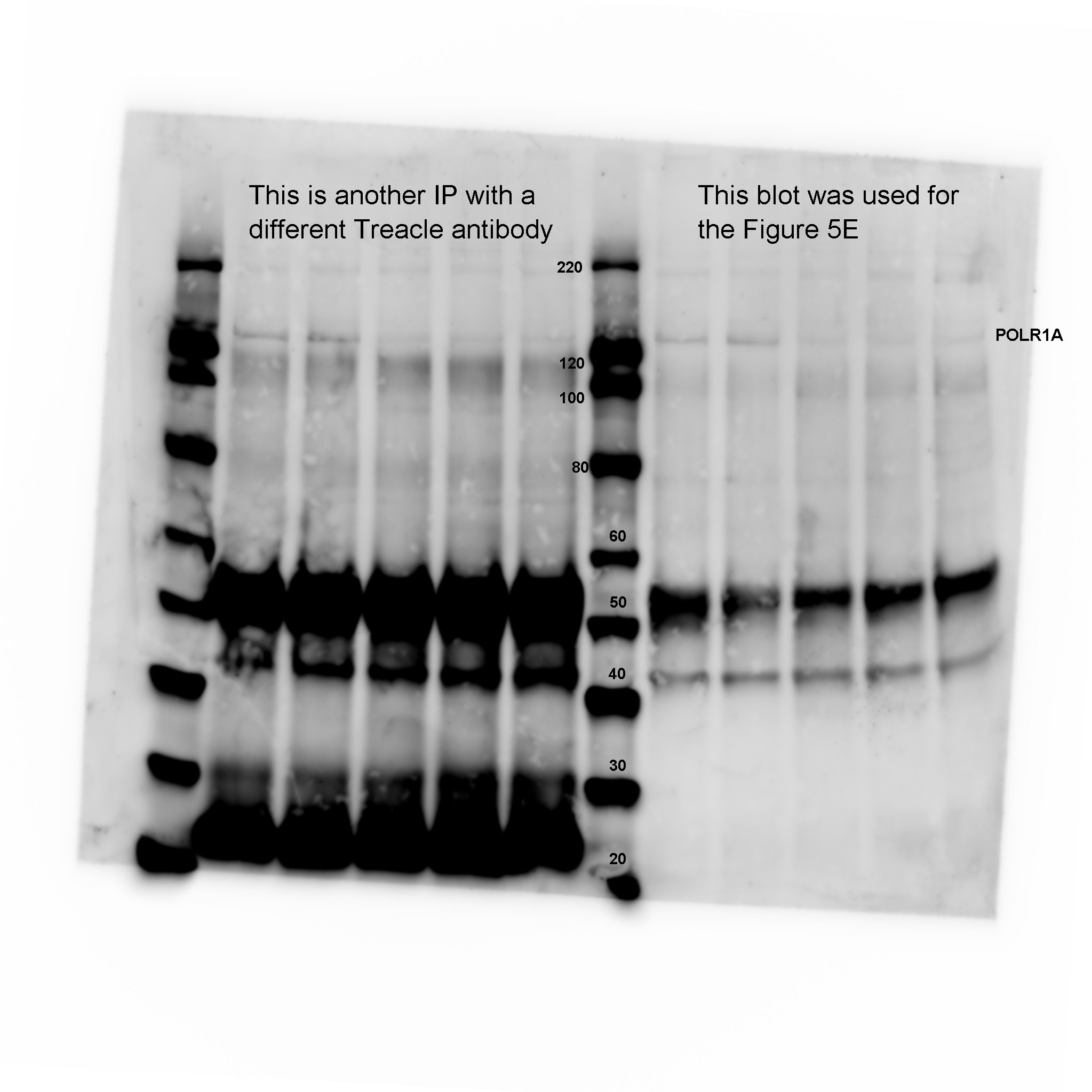

Supplement: Figure 5—source data 2. [file elife-88799-fig5-data2.zip › Figure 5-source data2/Figure 5-source data 5E/TCOF IPs POLR1A blot 2.5 min-annotated.jpg]

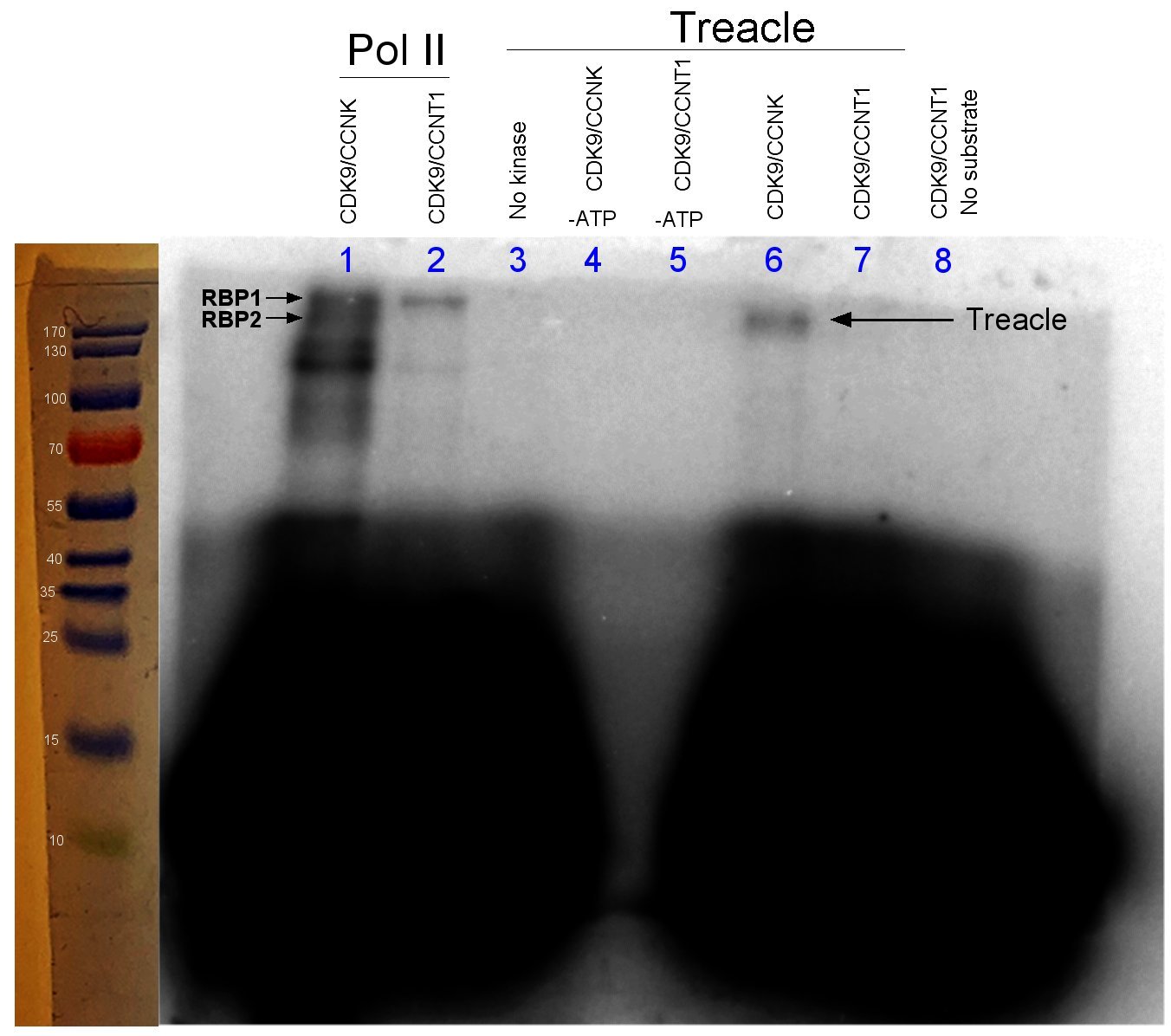

Supplement: Figure 5—figure supplement 1—source data 1. [file elife-88799-fig5-figsupp1-data1.zip › Figure 5 figure supplement 1-source data 1/B/Assay with ladder annotated.jpg]

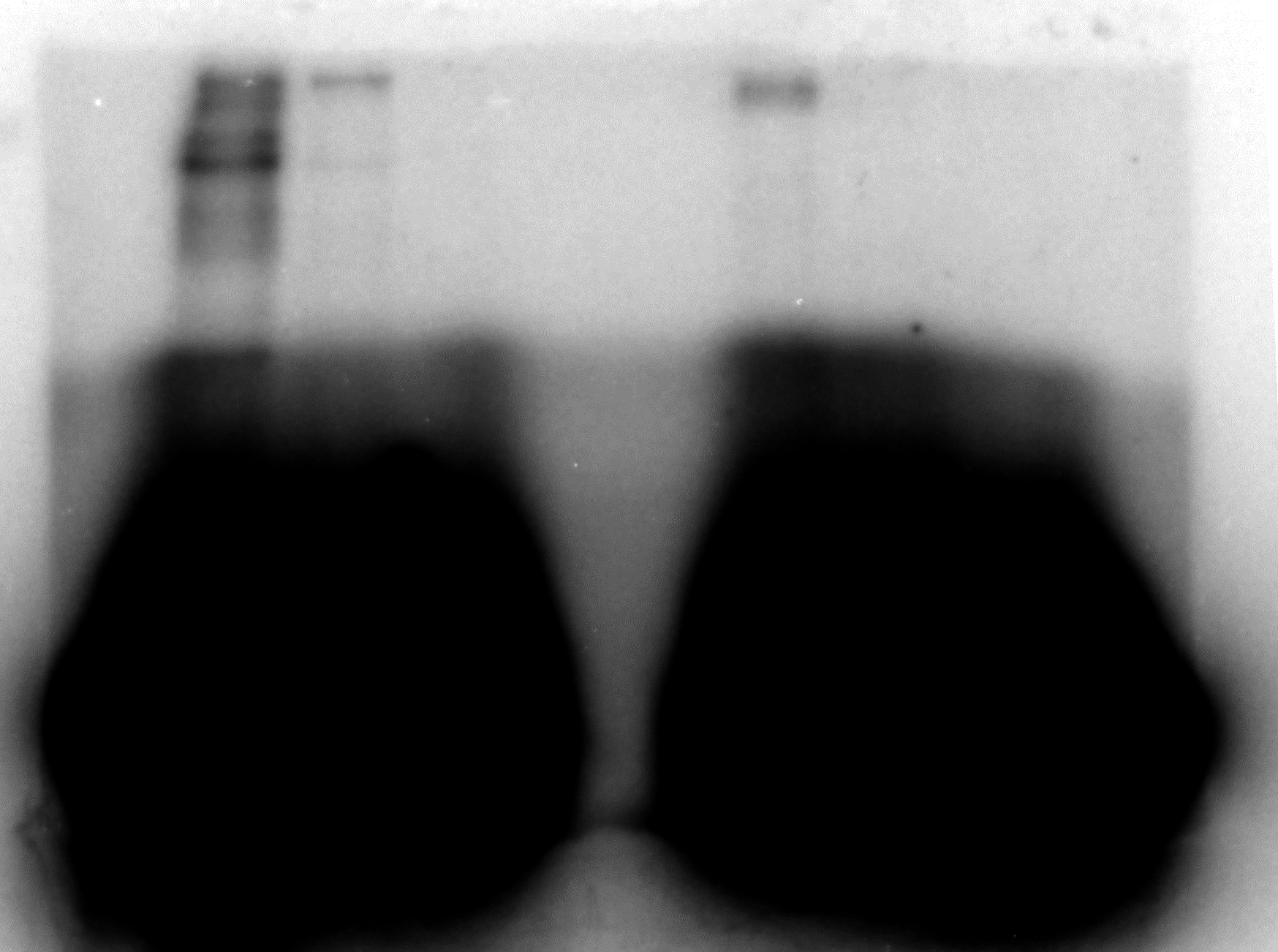

Supplement: Figure 5—figure supplement 1—source data 1. [file elife-88799-fig5-figsupp1-data1.zip › Figure 5 figure supplement 1-source data 1/B/CDK9 and cyclins T and K assays with Coomassie.tiff]

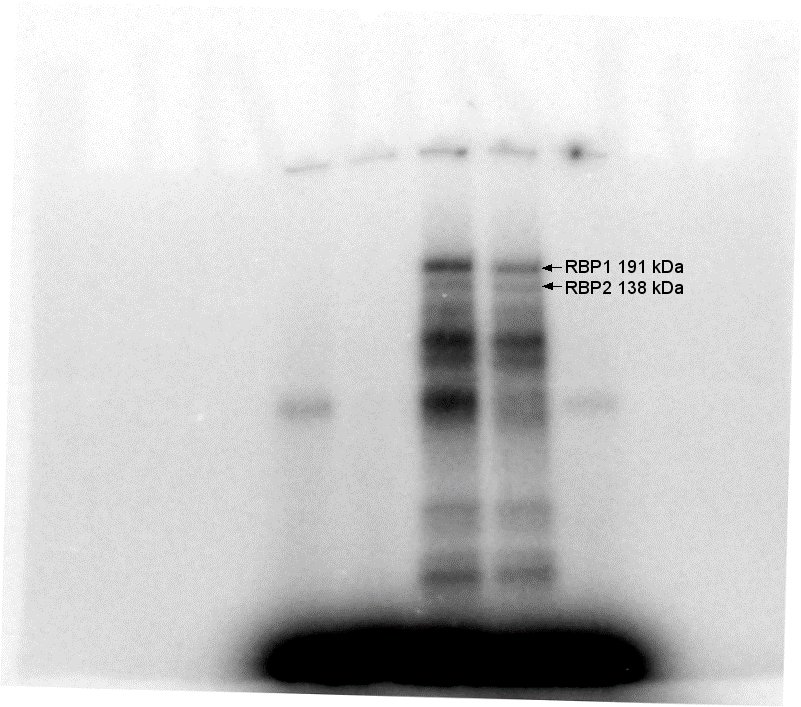

Supplement: Figure 5—figure supplement 1—source data 2. [file elife-88799-fig5-figsupp1-data2.zip › Figure 5 figure supplement 1-source data 2/C/CDK9 assay with PolI and Pol II annotated.jpg]
